# TAVI-in-homograft (TiH): open transcatheter aortic valve replacement in calcified aortic homograft case reports

**DOI:** 10.1186/s13019-019-1036-2

**Published:** 2019-11-27

**Authors:** Marco Gennari, Ilaria Giambuzzi, Gianluca Polvani, Marco Agrifoglio

**Affiliations:** 1 Centro Cardiologico Monzino IRCCS, Via Parea 4 – 20138, Milan, Italy; 2 Department of Cardiovascular Sciences and Community Health, University of Milan, Milan, Italy

**Keywords:** Homograft, Transcatheter aortic valve replacement, Redo surgery

## Abstract

**Background:**

Redo surgery in patient who underwent aortic valve replacement with an aortic homograft can result technically challenging because of the massive calcification of the conduit.

**Case presentation:**

We present a case of a patient who underwent open surgery on cardiopulmonary bypass assistance to implant a standard transcatheter aortic bioprosthesis through aortotomy in an off-label procedure and we discuss its safety and feasibility.

**Conclusions:**

The combination of open cardiac surgery and open trans-aortic implant of a transcatheter prosthesis may reduce the surgical risk shrinking the technical difficulties that the implantation of a standard surgical prosthesis would have given.

## Introduction

Surgical aortic valve replacement (SAVR) with a stentless root such as homografts may offer some clinical advantages, like improved hemodynamics performances and enhanced positive remodeling of the left ventricle [1], no need for lifelong anticoagulant therapy and relative low risk of endocarditis. However, these conduits are subjected to major deterioration, up to 94% at 10 years [2]. The risk of re-operation can be very high [3] because of massive calcification of the prosthesis and root that increases the complexity of the surgery, sometimes making replacement also of the conduit necessary. Open off-label transcatheter aortic valve replacement (TAVR) can be considered an interesting alternative to surgical replacement [4].

Hereby we present a case of a male patient who underwent what we named TAVI-in-Homograft (TiH) procedure.

## Case report

A 75-years-old Caucasian male was admitted to our clinic with a mixed aortic valve pathology. He had a positive cardiovascular anamnesis, since he underwent aortic valve replacement with a 27 mm size aortic homograft twenty-two years earlier.

Since then he, performed yearly echocardiographic follow-ups. The first signs of homograft degeneration presented 12 years after the implant with moderate aortic regurgitation and fibro-sclerosis of the aortic wall.

Ten years later there was evidence of worsening of aortic regurgitation in association with moderate stenosis (trans-valvular mean gradient 36 mmHg and maximum velocity of 3,3 m/s).

After 8 months from the last instrumental evaluation the patient went to the emergency department because of new onset dyspnea and fever. A new trans-thoracic echocardiogram showed worsening of the left ventricular ejection fraction (EF 42%), new evidence of left ventricle dilation with an end-diastolic volume (EDV) of 245 ml and vegetation on the aortic leaflets. Blood cultures were positive for *C. hominis,* which lead to the diagnosis of endocarditis and specific antibiotic therapy was started with Meropenem and Ceftriaxone. After three weeks of therapy, the inflammatory markers were negative, the clinical aspects of the patient were ameliorated and the echocardiogram showed improvement of the EF (52%) and positive remodeling of the left ventricle (EDV 161 ml) while vegetations and severe aortic regurgitation were confirmed, and echocardiographic aortic stenosis parameters worsened (trans-valvular mean gradient 58 mmHg and maximum velocity 5 m/s). Echocardiographic and CT scan images did not show any image suggestive of abscess.

Because of the frailty of the patient, the complexity of a redo-surgery in the setting of an extremely calcified and dilated homograft the heart team proposed an implantation of a transcatheter aortic valve on cardiopulmonary bypass (CPB) through median sternotomy.

**Fig. 1:**
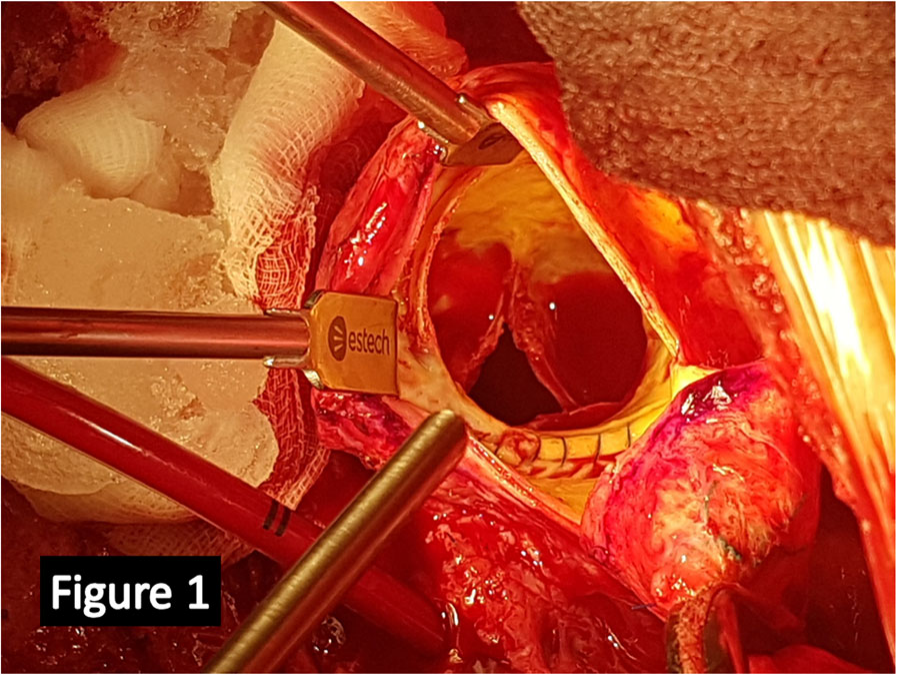
Intraoperative picture of the degenerated homograft. Notice the calcified leaflet and their fixed opening position

**Fig. 2:**
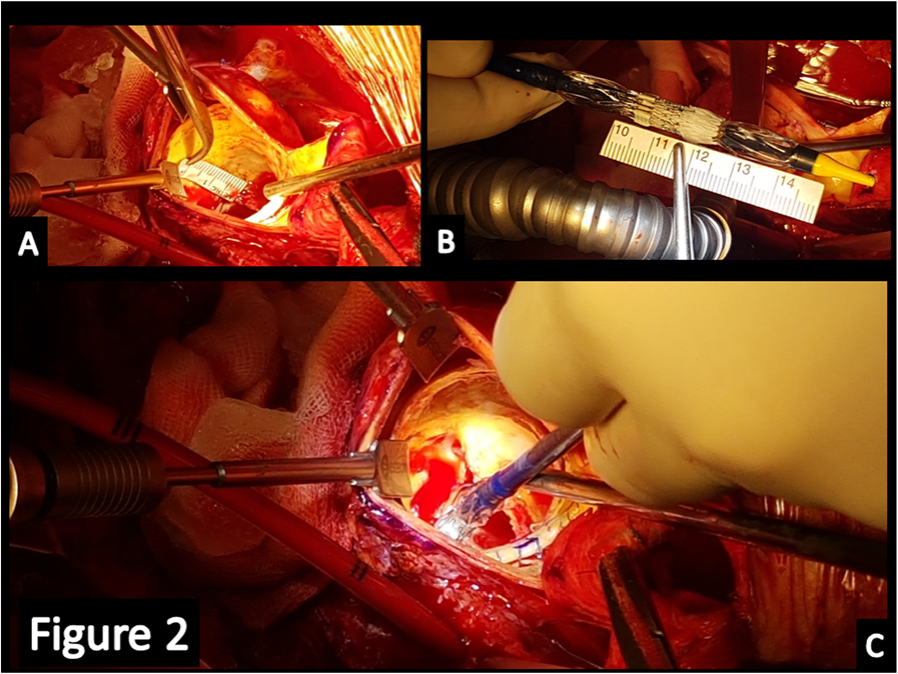
We measured the virtual basal ring to coronary ostia height (**a**) and we compared them with the prosthesis height (**b**). Finally, we deployed the valve at the most convenient point (**c**)

**Fig. 3:**
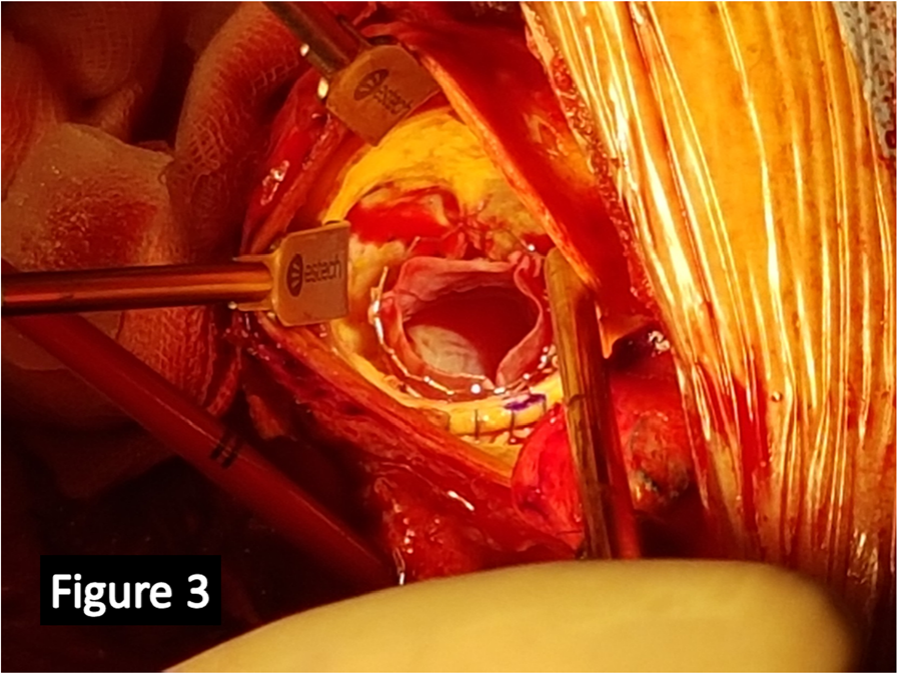
Intraoperative picture of the final result. Once deployed we explored the prosthesis boundary and we judged the sealing satisfactory. No post-dilatation was needed in this case

Femoro-femoral CPB was established and median re-sternotomy performed. The technique was as follow:

- A small aortotomy was performed 2 cm above the calcified and dilated homograft and then the leaflets were explored. The leaflets appeared retracted and heavily calcified (Fig. 1), and permitted only the passage of an Hegar sized 19 mm. Small pieces of the homograft were collected to be cultured
- the degenerated leaflets were removed, after careful evaluation and confirmation of absence of abscesses, and the height of the coronary ostia measured (Fig. 2a); the height of the main left was 9 mm while that of the right coronary ostium was 10 mm and these measurements were compared with the height of the transcatheter prosthesis (Fig. 2b). Then the deployment at nominal volume of a #23 Sapien 3 (*Edwards Lifesciences, Irvine, USA*) balloon-expandable prosthesis was performed under direct view (Fig. 2c)
- at the end we noticed a satisfactory expansion of the prosthesis-in-homograft (Fig. 3) so we did not perform any post-dilatation
- finally thromboendoarterectomy of the degenerated homograft was performed to remove the most calcified parts and to allow direct closure with Proline 4–0. The weaning from CPB was quick and easy.

The following in-hospital course was uneventful, the homograft biopsies were negative and the post-operative echocardiogram showed only a mild residual aortic regurgitation (partially para-valvular). The patient was discharged in the 7th post-operative day.

After 14 months from the procedure the patient is alive and well, asymptomatic, and the residual mild aortic regurgitation is stable.

## Discussion

Valvular and vascular tissues from a cadaveric donor are often utilized in congenital malformation and in the adult in the setting of acute endocarditis due to their more likely resistance to the infections.

We previously report other cases of severe calcified degenerated homograft that needed other than surgical replacement due to the impossibility to perform secure decalcification [5]. The trans-aortic route, introduced in 2012 by Bapat [6], has been proved to be safe when patients are correctly selected [7, 8].

Trans-aortic TAVR was chosen after exclusions of the sutureless valves. First of all, we could not use the Intuity prosthesis because of the impossibility to place any stitches on the homograft. The other possible choice was the Perceval prosthesis, but we avoided it because of the higher profile of the prosthesis (its stented structure would have reached over the homograft, which was anyway dilated, and the support on the ascending aorta would have been less table) and because of the higher and larger aortotomy that it would have needed. Moreover, any peripheral approach was excluded to avoid the need of a wire, that could have easily teared up the spotted calcifications expanding from the homograft to the ascending aorta. Instead, a trans-apical approach has been excluded because of the low EF.

In such cases a TAVI-in-Homograft (TiH) procedure may be the only reasonable possibility to overcome the anatomical finding discovered at the operating theatre, as also other groups [9, 10] have described.

In this case we have presented the heart team evaluated the frailty of the patient and the anatomy of the homograft. The graft was so calcified that a standard surgery requiring stitches would have been technically impossible, but it would have also made very dangerous a standard transcatheter procedure because of the aortic calcification [11, 12] and the poor control upon releasing the balloon-expandable prosthesis.

## Conclusion

The combination of open cardiac surgery with CPB and open trans-aortic implant of a transcatheter prosthesis decreased the surgical risk and permitted to perform the procedure safely, shrinking the technical difficulties that the implantation of a standard surgical prosthesis would have given.

Larger series and studies on this particular subpopulation of patients are warranted to drive final conclusion.

## Data Availability

Data and materials are available.
